# Periprosthetic Joint Infection: Modern Diagnostic Markers and Their Limitations

**DOI:** 10.7759/cureus.100329

**Published:** 2025-12-29

**Authors:** Adeolu Badejo, Michael O Kolade, Abiodun C Adegbesan, Kenechukwu Igbokwe, Imri Adefokun, Adedayo Adebayo, Funbi Ayeni

**Affiliations:** 1 Orthopaedic Surgery, Royal Victoria Infirmary, Newcastle upon Tyne, GBR; 2 General Surgery, Scarborough General Hospital, Scarborough, GBR; 3 Global Health, Menzies Institute for Medical Research, University of Tasmania, Hobart, AUS; 4 Trauma and Orthopaedics, Gateshead Health NHS Foundation Trust, Gateshead, GBR; 5 Orthopaedic Surgery, Bowen University Teaching Hospital, Ogbomoso, NGA; 6 Trauma and Orthopaedic Surgery, University College Hospital, Ibadan, NGA; 7 Trauma and Orthopaedics, Imperial College Healthcare NHS Trust, London, GBR

**Keywords:** alpha-defensin, diagnostic biomarkers, periprosthetic joint infection (pji), synovial fluid analysis, total hip arthroplasty

## Abstract

Joint arthroplasty (JA) is a common surgical procedure that provides significant relief from degenerative joint pain, notably increases quality of life for patients, and markedly improves functional status. However, despite its many benefits, JA is not without risk, and one of the greatest risks patients who undergo JA face is periprosthetic joint infections (PJIs). PJIs are defined as infections occurring after the implantation of an artificial joint and have been shown to result in substantial morbidity, high treatment costs, and increased long-term mortality. Despite improvements in diagnostic criteria over time, early and accurate diagnosis of PJIs continues to be challenging. Challenges include overlapping clinical symptoms, a range of potential host responses to infection, and the absence of a single definitive diagnostic marker. The purpose of this narrative review is to evaluate the various diagnostic markers currently available for use in assessing PJI. A comprehensive search and review of all systematic reviews, meta-analyses, and diagnostic accuracy studies was done. While traditional serum inflammatory markers, such as C-reactive protein (CRP) and erythrocyte sedimentation rate (ESR), can remain useful in identifying infections, their sensitivity is significantly lower in cases of chronic or low-grade infections. Synovial fluid biomarkers, such as leukocyte esterase, alpha-defensin, and calprotectin, have demonstrated improved diagnostic performance when compared to traditional inflammatory markers. These markers appear to have superior ability to rule in or rule out infection; however, cost, variability in threshold values, and susceptibility to confounding variables limit their widespread adoption. Molecular diagnostics, including polymerase chain reaction (PCR) and next-generation sequencing (NGS), represent potentially powerful tools for detecting low-virulence and culture-negative organisms; however, these tools remain limited by cost, laboratory requirements, and interpretive difficulties. The evidence supports a multi-modal approach to diagnosing PJI. This approach would utilize a combination of clinical assessment and established serum and synovial markers, along with the selective use of advanced biomarkers and molecular assays. Future advancements in diagnosing PJI depend on several factors including the development of standardized diagnostic thresholds, improvement in the affordability and accessibility of point-of-care tools, and validation of artificial intelligence (AI)-enhanced diagnostic models capable of utilizing large amounts of complex data to increase diagnostic confidence and ultimately lead to better patient outcomes.

## Introduction and background

Periprosthetic joint infection (PJI) is currently considered one of the most serious complications encountered in modern arthroplasty [1]. With the exponential growth of total/partial joint replacement surgeries worldwide, there has also been a commensurate increase in the frequency of PJIs [1]. In addition to the increased hospital stay and readmission rates following PJI diagnosis, reoperations are commonly required, resulting in longer periods of postoperative recovery and potentially poor functional outcomes [2]. Further complicating matters, individuals with PJI have poorer long-term joint function, lower quality of life, and greater levels of psychological distress when compared with their counterparts without infection [2]. A recent systematic review and meta-analysis of 19,917 patients demonstrated a three-year mortality rate of 11% post-PJI after primary total hip arthroplasty, a statistic that is similar to the five-year survival rates of some of the more common types of cancer such as breast or prostate cancer [2]. Also, the economic consequences of PJI are substantial, with projected US treatment-related expenditures exceeding $750 million by 2030 alone [2]. Therefore, these findings emphasize the urgent necessity for the development of rapid and accurate methods for diagnosing PJI.

Because many of the clinical signs of PJI can be similar to those seen with aseptic loosening, inflammatory arthritides, and early postoperative inflammation, diagnosing PJI can be problematic [3]. As no single diagnostic test has demonstrated adequate sensitivity and specificity to serve as a single diagnostic tool, clinicians must rely upon the use of composite criteria and multi-modal assessments to diagnose PJI [3]. Additionally, several different diagnostic approaches and thresholds have been proposed by different organizations (e.g., the Musculoskeletal Infection Society (MSIS), the International Consensus Meeting (ICM), and the European Bone and Joint Infection Society (EBJIS)) to diagnose PJI; therefore, differences in diagnostic classifications exist between studies and among various clinical settings [2].

The objective of this narrative review is to provide a synthesis and critical appraisal of the current literature regarding serum, synovial, and molecular diagnostic markers used to evaluate PJI. Another goal of this review is to provide clinicians with an understanding of the strengths and limitations of each diagnostic modality to support the use of evidence-based decision-making in clinical practice.

## Review

Overview of diagnostic frameworks

Current diagnostic methods for diagnosing PJI are based upon a set of organized frameworks which include the integration of clinical, serologic, synovial, microbiologic, and intraoperative data using various weighted criterion systems. The MSIS definition was developed at the first ICM, and these definitions have been the most commonly referenced frameworks since their development, with most current diagnostic accuracy studies using MSIS as the reference standard for comparison in large-scale meta-analyses of synovial α-defensin and leukocyte esterase (LE) [4]. The MSIS framework uses both major and minor criteria to evaluate data collected during the evaluation process. Major criteria (such as the presence of a sinus tract or two positive cultures) provide definitive diagnostic value, whereas minor criteria (such as increased serum inflammatory markers, increased white blood cell count in the synovial fluid, and certain biomarkers) contribute to a cumulative diagnostic threshold [4].

In addition to expanding diagnostic inclusiveness, the EBJIS 2021 criteria also identified a "likely infection" category that will increase the sensitivity of classifying PJIs when compared to the MSIS major criteria [4]. The EBJIS criteria emphasize both synovial analysis and microbiology, and there has been a growing body of evidence demonstrating that the use of synovial-based tests provides better exclusion of infection than the use of serum markers [5].

All frameworks agree that clinical suspicion should be the primary method of evaluating potential PJIs since no individual biomarker or imaging study has the ability to provide sufficient diagnostic information alone. While imaging modalities like positron emission tomography/computed tomography (PET/CT) may provide additional information for supporting diagnosis, multiple systematic reviews and meta-analyses demonstrate heterogeneity in the performance of imaging modalities across the spectrum of conditions characterized by inflammation and infection and, thus, limited ability to differentiate between infection and aseptic inflammation [6]. Therefore, frameworks emphasize the need for a comprehensive and multi-modal evaluation rather than relying on the results of any one parameter or test.

Serum-based traditional biomarkers

C-reactive Protein (CRP)

CRP is synthesized by hepatocytes in response to IL-6-mediated systemic inflammation and is frequently used as a serum screening biomarker for patients who are suspected to have PJI. Although CRP has become an integral component of both MSIS and ICM minor criteria for diagnosing PJI, recent reviews have noted that the diagnostic performance of CRP is highly variable, especially for indolent or low-grade infections, where the sensitivity of CRP is significantly reduced [3,4]. Additionally, postoperative increases in CRP due to normal physiological inflammation postoperatively limit the specificity of CRP for diagnosing PJI and make it unreliable during the early postoperative time frame [3,4].

Erythrocyte Sedimentation Rate (ESR)

Although ESR has been utilized historically as an inflammatory marker, it currently holds greater relevance for identifying chronic PJI rather than acute PJI. Despite this historical role, ESR lacks specificity for PJI because other conditions, including rheumatoid arthritis, chronic inflammatory disease, and age-related changes, can independently cause elevations in ESR, thereby complicating the interpretation of ESR in the context of assessing PJI [4]. Like CRP, ESR is typically employed in conjunction with synovial or microbiologic evaluations for assessing PJI, rather than as a definitive diagnostic marker.

D-dimer

The D-dimer assay can serve as an adjunct biomarker due to the relationship between infection-induced systemic coagulation activation and fibrinolysis [5]. The evidence for D-dimer's role in diagnosing PJI is however mixed. Although several meta-analyses have shown that D-dimer's sensitivity and specificity vary greatly among populations studied, many reported that false-positive values are present among patients experiencing postsurgical hypercoagulable states, those with malignancies, or those with thrombosis [5,7,8]. A recent comparison of D-dimer and serum fibrinogen levels suggested that serum fibrinogen was superior to D-dimer for the detection of PJI, yielding better diagnostic accuracy [8].

Synovial fluid markers

Synovial White Blood Cell Count and Polymorphonuclear Neutrophil Percentage (PMN%)

The white blood cell count and PMN% are core indicators and are part of each of the established diagnostic criteria. These are useful because they directly indicate the level of inflammatory reaction in the joint space and they are demonstrated to be moderately accurate in diagnosing PJI when compared to the MSIS reference standards [4]. However, the best threshold to apply varies between hip and knee arthroplasties as well as between chronic and acute PJI, thereby limiting its application to a specific population [9]. Furthermore, these are less accurate in cases involving inflammatory arthropathy, crystal diseases, and postoperative conditions; in these conditions, the increased white blood cell counts observed in the synovial fluid could be attributed to non-infectious inflammation [4].

Culture

Fluid obtained from the aspiration of the infected prosthetic joint space is the most commonly used method for identifying microorganisms responsible for the infection of the joint. However, this method has limited sensitivity for detecting certain low-virulent microorganisms, for identifying organisms isolated from cultures taken after antibiotics were administered, and for detecting organisms within biofilms. Therefore, many instances of PJI result in false negatives [10]. Additionally, slow-growing microorganisms often delay the ability to definitively identify the causative organism; thus, there is an increasing need for additional rapid methods to detect biomarkers of infection.

LE Rapid Strip Test

The LE strip test is a point-of-care method for rapidly determining if there are neutrophil-derived esterases present in the synovial fluid. The results of the meta-analysis of the diagnostic accuracy of the LE strip test revealed high specificity (96%), but intermediate sensitivity (79%) for PJI, indicating that this test could be used as a preliminary screening method [4]. However, limitations associated with the use of this test include subjective color interpretation (i.e., "borderline" color interpretations) and interference from blood in the synovial fluid which compromises the diagnostic reliability of the test [4].

Alpha-Defensin

Alpha-defensin is a neutrophil-derived antimicrobial peptide released into the synovial fluid in response to the presence of bacteria. Studies have demonstrated that alpha-defensin is highly sensitive and specific for the diagnosis of PJI, with pooled sensitivity and specificity being 87% and 97%, respectively [4]. Both enzyme-linked immunosorbent assay (ELISA) and lateral flow tests are widely utilized to evaluate alpha-defensin; however, ELISA appears to be slightly more accurate than lateral flow tests [11]. Although alpha-defensin testing has advantages, it is very expensive, there are false-positive results in cases of metallosis and/or severe inflammation, and not all medical facilities offer this type of testing [4].

Calprotectin

A new synovial biomarker that reflects neutrophil activation, calprotectin, is an increasingly promising alternative to alpha-defensin. Results from several meta-analyses show that calprotectin has high specificity and moderate-to-high sensitivity for the diagnosis of PJI and that at least one study showed similar diagnostic performance to alpha-defensin despite significantly lower costs [12]. Variability in the assays used and variable thresholds in the studies limit the consistency in the accuracy of PJI diagnosis using calprotectin, and therefore, there is a need for the standardization of both the assays and the thresholds [12].

Tissue-based and molecular diagnostics

Histopathology

Histopathology, specifically intraoperative pathology and especially frozen-section pathology, has traditionally served as a small part of PJI diagnosis in the form of a minor diagnostic component of both the MSIS and ICM criteria, due to its well-established historical use as a diagnostic tool [3]. Frozen-section pathology allows the pathologist to quickly assess whether there is significant neutrophilic inflammation within the periprosthetic tissue, allowing for rapid intraoperative decision-making to determine if this represents a possible infection versus a mechanical issue related to the prosthetic device [3]. Despite its historic use as a diagnostic tool for PJI, histopathology has several drawbacks that limit its use as a definitive stand-alone test for PJI diagnosis. The greatest drawback of histopathology is the considerable amount of variability among operators/pathologists that exist in terms of how they evaluate samples for the presence of neutrophils and how many neutrophils must be present before it can be said that there is likely an infection [3]. This variability creates an environment where it is difficult to compare data among centers/studies regarding the diagnostic accuracy of histopathology for PJI. Histopathology, therefore, does not lend itself to being used as a definitive stand-alone test due to its inability to be reliably compared among various centers/studies.

Next-Generation Sequencing (NGS)

Recent advances in NGS have made it a rapidly evolving technology that has improved our ability to detect low-virulent pathogens that are oftentimes missed using standard microbiological culturing techniques [13]. These include such bacteria as *Cutibacterium acnes* and other gram-positive organisms that are known to grow slowly in vitro [13]. Since NGS sequences the DNA of microorganisms directly from either synovial fluid or tissue samples, it is able to overcome some of the limitations associated with culturing and provide a more complete picture of what types of organisms are present in these types of samples, especially in cases where traditional culture-based methods are unable to identify causative organisms [13]. Systematic reviews have shown that NGS has a greater sensitivity than traditional culture methods for identifying the causative organisms in patients diagnosed with PJI [13,14]. However, despite its advantages over traditional culture methods, NGS has its own set of limitations. It is currently expensive, requires specialized equipment and personnel to operate, and takes longer to obtain results compared to traditional culture methods [13]. Another limitation of NGS is the potential for contamination from environmental organisms and/or commensal organisms that may be present on the skin/surfaces of surgical instruments that may be introduced into the sample during collection and processing. This potential for contamination could result in the false-positive identification of organisms that are not actually contributing to the patient's condition [13].

Polymerase Chain Reaction (PCR)

PCR-based tests represent another method for identifying bacterial DNA and are commonly used in conjunction with traditional culture methods for diagnosing PJI [7]. Synovial fluid PCR has increased the number of diagnoses in patients who have received antibiotics prior to the collection of their synovial fluid since antibiotic treatment would reduce the number of viable bacteria present in the culture, thus reducing the likelihood of obtaining a positive culture result [7]. Additionally, systematic reviews have demonstrated that the sensitivity of PCR-based tests for detecting the causative organisms of PJI is significantly higher than those obtained using traditional culture methods [7]. However, similar to NGS, PCR-based tests also suffer from significant limitations. One major limitation is the propensity for false positives due to the presence of non-viable bacteria or contaminants, which are undistinguishable from viable bacteria by PCR [7]. Another major limitation of PCR-based tests is that PCR alone lacks the capability to distinguish between colonization/contamination and true infection [7] which severely reduces its specificity when utilized as a single test for the diagnosis of PJI.

Emerging biomarkers and technologies

*Machine Learning and Artificial Intelligence* (*AI)-Assisted Diagnosis*

One technology being researched is machine learning, specifically AI-assisted diagnosis. Early studies have used machine learning to develop predictive algorithms using a combination of biomarkers, including CRP, ESR, leukocytes in the synovial fluid, and various molecular metrics, to diagnose PJIs [3].

The use of multi-dimensional datasets to identify patterns has allowed researchers to potentially provide a method to overcome the limitations associated with the use of individual parameter diagnostics [3]. Although early results have shown great promise, the practicality of this technology is limited because it requires a large, high-quality dataset, as well as external validation across diverse patient populations; however, this has proven difficult to achieve [3].

Metabolomic and Proteomic Markers

Metabolomic and proteomic technologies have also drawn initial attention due to their potential ability to detect host-pathogen interactions and provide metabolomic and/or proteomic signatures unique to PJIs [3]. Biomarkers identified through metabolomic and proteomic testing represent preliminary findings and currently lack validated clinical thresholds, are poorly reproducible, and lack large-scale studies supporting their clinical utility [3].

Point-of-Care Diagnostic Devices

Point-of-care diagnostic devices have grown significantly and include LE strip tests, calprotectin dipstick assays, and other emerging handheld devices for analyzing the synovial fluid intended to enhance intraoperative or clinic-based PJI diagnosis [4]. Both LE and calprotectin-based devices provide rapid, inexpensive assessments of patients with potential PJI while achieving higher sensitivity than traditional serum markers [5].

Despite the benefits of LE and calprotectin-based devices, they are limited in their adoption by variability in the performance of different devices, possible interference caused by blood-contaminated joint aspiration samples, and varying availability of the devices at hospitals and centers [4].

Limitations Across All Diagnostic Markers

No single diagnostic marker for PJI is sufficiently accurate to be relied upon as an independent indicator of infection; therefore, clinicians will continue to employ a multi-modal diagnostic approach [3]. In addition to chronic disease states, such as rheumatoid arthritis, gout, metabolic syndrome, and obesity, which can increase levels of systemic inflammatory and synovial markers, thereby lowering the specificity of diagnostic tests [5], comorbid conditions may confound diagnostic interpretation. Due to the expected elevation of serum markers, such as CRP, ESR, and D-dimer, after surgery, clinicians cannot rely on serum markers to assess PJI during the early postoperative period [4]. Furthermore, there is considerable variation in diagnostic thresholds reported in studies, especially for synovial white blood cell count, PMN%, D-dimer, and calprotectin, which limits comparability between studies and standardization of guidelines [9].

Also, high-cost assays, including those utilizing alpha-defensin (especially ELISA platforms) and NGS, limit access to these assays in many healthcare systems and restrict their adoption into routine clinical practice [4]. Finally, advanced molecular diagnostics require specialized laboratory equipment and trained personnel, which further limits their availability in routine clinical practice [13].

## Conclusions

Despite the emergence of novel molecular testing and biomarkers for PJI, clinical diagnosis of PJI is still a significant challenge. Infections caused by diverse pathogens can lead to varying degrees of host response, patient comorbid conditions, and postsurgical changes that obscure signs of an infection. Hence, conventional markers, such as CRP, ESR, etc., have poor performance in patients with chronic or mild infections. New biomarkers in the synovial fluid (calprotectin and alpha-defensin) and molecular diagnostics (PCR, NGS) offer enhanced sensitivity and identify patients whose cultures may be negative; however, these new diagnostics are limited due to the costs associated with them, their accessibility, the variability of the threshold values used, and the interpretative complexity associated with them. The current literature supports a multi-modal clinician-driven method of diagnosing PJI. Future improvements are anticipated based on the development of standardized thresholds, greater availability of new biomarkers, and validated AI-based methods for the clinical diagnosis of PJI.
